# Effects of saturated fat supplementation and forage-neutral detergent fiber content on production performance of lactating buffaloes

**DOI:** 10.5713/ab.24.0760

**Published:** 2025-04-04

**Authors:** Saba Anwar, Anjum Khalique, Hifzulrahman, Muhammad Naeem Tahir, Muhammad Asim Tausif, Burhan E Azam, Hina Tahir, Sundas Qamar, Muneer Ahmad Khan, Ijaz Hussain, Muhammad Naveed-ul-Haque

**Affiliations:** 1 Buffalo Research Institute, Kasur, Pakistan; 2 Department of Animal Nutrition, University of Veterinary and Animal Sciences, Lahore, Pakistan; 3 Department of Livestock Production, University of Veterinary & Animal Sciences, Lahore, Pakistan; 4 Department of Livestock Management, The Islamia University Bahawalpur, Bahawalpur, Pakistan; 5 Livestock Experiment Station, Pattoki, Pakistan; 6 Department of Livestock and Dairy Development, Lahore, Pakistan

**Keywords:** Buffalo, Forage-neutral Detergent Fiber (fNDF), Methane, Milk Fat Content, Saturated Fat

## Abstract

**Objective:**

The study aimed to find out the effects of dietary forage-neutral detergent fiber (fNDF), saturated fat, and their interaction on milk production, composition, and blood metabolites in lactating buffaloes.

**Methods:**

Sixteen multiparous buffaloes received 4 treatments with 2 different levels of fNDF and saturated fat according to a changeover design with 6-wk periods under restricted feed intake. Within each fNDF level, the buffaloes received 1 of the 2 saturated fat levels for 3-week subperiods, which corresponded to the following treatments: 1) 23.4% fNDF and 0% fat, 2) 23.4% fNDF and 2% fat, 3) 33.5% fNDF and 0% fat and 4) 33.5% fNDF and 2% fat.

**Results:**

Increasing fNDF levels increased the body condition score (BCS), body weight (BW), and rumen pH. Despite these changes, levels of fNDF did not alter the milk components and yield. Dietary saturated fat supplies improved milk fat content and tended to enhance the yields of milk fat and fat-corrected milk. In addition, the saturated dietary fat supplies increased BW, milk nitrogen efficiency, and cholesterol concentration, and decreased predicted methane yield. However, the milk yield, BCS, and rumen pH remained unaffected by dietary fat supplies. A high fNDF diet with dietary saturated fat supplementation tended to increase the milk fat content.

**Conclusion:**

Balancing diets with saturated fats and fiber significantly affects productivity. These results indicate that feeding fNDF with saturated fat may be a viable option for producers to improve milk fat production, enhance body condition, and reduce methane production in buffaloes.

## INTRODUCTION

Water buffaloes (*Bubalus bubalis*) are ranked 2^nd^ by contributing 15% to the worldwide milk supply annually [1]. The herd average milk production of buffalo is less and can be improved through nutritional manipulation [2,3]. One way to increase buffalo milk production is through concentrate feeding, but its rapid fermentation and overconsumption can lead to acidosis. The alternative method is fat supplementation, but feeding fat above the recommended level can impede ruminal fiber digestion [4]. Among the dietary fats, saturated fatty acids have less bad effects on rumen fiber digestion and increase milk yield and fat in buffaloes [2]. Saturated fat affects energy partitioning depending upon the forage neutral detergent fiber (fNDF) content of the diet from 21 to 120 days postpartum in dairy animals [5].

Feeding a high fiber diet in buffalo farming is a common practice. It is efficiently utilized by buffaloes [6] due to a higher population of cellulolytic bacteria and fungal zoospores. According to NRC [4] at least 75% of dietary NDF in the diet of dairy animals should come from forages to ensure optimal rumen function. Buffalo milk has a higher fat content compared to cow’s milk [7], which can be further increased through dietary fiber supplementation [8] and fat feeding [2]. The replacement of dietary fiber with starch [9] and fat supplementation [10] reduces methane production in ruminants. Additionally, a high-forage diet supports fat utilization by maintaining normal rumen function and facilitating fat absorption. As a result, forages and fat complement each other by enhancing energy intake and rumen function.

The interaction of fat and fiber has been well reported in dairy cows [5,11] but limited information is available on buffaloes. Therefore, the present study was designed to determine the independent and interactive effects of saturated fat with fNDF on the productive performance of buffaloes.

## MATERIALS AND METHODS

### Animals

The study was performed at a Livestock Experiment Station (LES) in Bhunikey, Punjab, Pakistan from May to August 2022. The study was conducted according to ethical rules and regulations approved by a committee of dairy section LES Bhunikey, for the use and welfare of experimental animals vide letter No. 894 dated 30.04.2022. Sixteen multiparous lactating buffaloes, with (mean±standard deviation) 6.04±0.92 kg/d of milk yield, 5.70±0.64% of milk fat, 519±44.78 kg of body weight (BW), and 118.44±38.92 days in milk were enrolled.

### Study design, treatments, and feeding

The 4 dietary treatments were arranged in a 2×2 factorial design with 2 levels of forage NDF and 2 levels of saturated fat. The buffaloes received either a low or high fNDF diet (23.42% or 33.54% fNDF) according to a changeover design with a 6-wk period (macro period) for each level. Each 6-wk period (macro period) was further divided into 2 sub-periods of 3-wk (micro period) so that each buffalo received 1 of the 2 saturated fat levels (0% and 2% fat). The resulting treatments were: 1) 23.42% fNDF and 0% fat, 2) 23.42% fNDF and 2% fat, 3) 33.54% fNDF and 0% fat, and 4) 33.54% fNDF and 2% fat. The low (23.42%) and high fNDF (33.54%) levels were formulated by manipulating the proportions of wheat straw (from 29% to 42%) and concentrate (from 71% to 58%), respectively, whereas high fat (2% per kg diet dry matter [DM]) level was obtained by adding 300 g/d saturated fat supplement in top-dressed fashion (Saturated fat; ProLac-100, 99.68% fatty acid [FA]: 0.20% C18:0, 4.18% C18:1 & above, and 95% C16:0). The low fat treatment (0% per kg diet DM) had no fat supplementation. The duration of the study was 94 d along with the first 10 d of the adaptation period. There were 2 long periods which were referred to as the macro period of 42 days (d) with 2 micro periods of 21 d within each macro period. The dietary treatments were formulated using Cornell-Pen-Miner-Dairy 3.0.10 software based on Cornell Net Carbohydrate and Protein System version 5.0.2. These diets were offered at 09:00 am in total mixed ration form. The chemical composition and ingredients of diets are mentioned in Table 1.

### Sample collections and analysis

Each feed ingredient sample (concentrates and wheat straw) was taken twice in each period and composited for further analysis. These samples were measured for DM (method; 934.01), ether extract (method; 920.39), ash (method; 942.05), and crude protein (984.13, N×6.25; Kjeldahl method) according to International official methods of the AOAC [12]. The Ankom-2000 fiber analyzer (Fairport, NY, USA) was used for the analysis of acid-detergent fiber and NDF. The NDF provided from the wheat straw was the fNDF. The milk samples from all buffaloes were collected on alternate days in the first 2 weeks and every day in the last week of each period. An ultrasonic milk analyzer (Lactoscan S 1720; Milkotronic, Zagora, Bulgaria) was used for the evaluation of morning and evening milk samples separately. Blood samples were taken on the third last day (18^th^ day) of each period from the jugular vein. Heparinized syringes were used for blood sample collection. Blood was centrifuged at 4°C for 15 min at 2,000×g. The plasma was separated with micropipettes, aliquoted in 1 mL Eppendorf tubes, and stored at −20°C to analyzed by using commercially available enzymatic kits (Randox Laboratories, Crumlin, UK). The contents of glucose, cholesterol, and triglyceride were determined with a biochemical analyzer (RX Monza; Randox Laboratories). The BW and body condition score of experimental animals were recorded before the start and then at the end of each period before feeding. The samples for ruminal pH measurement were collected on the last day of each period according to Adin et al [13].

### Calculations

The non-fibrous carbohydrates were estimated by following NRC [4]. Protein-corrected milk (PCM), 4% fat-corrected milk (FCM), and energy-corrected milk (ECM) were measured by following Akhtar et al [3]. Gross efficiency of metabolizable protein (MP) = milk protein yield/MP intake, metabolic efficiency of MP = milk protein yield/(MP intake–MP for growth, pregnancy, and maintenance), and feed efficiency = milk yield/dry matter intake (DMI) were determined by following INRA [14]. Milk nitrogen efficiency (MNE), milk energy (MkE), and milk nitrogen (MkN) were measured by following Akhtar et al [3]. Methane intensity, production, and yield were determined with an equation reported by Patra [15].

### Statistical analysis

Data from each period were analyzed using the PROC MIXED procedure of SAS University Edition (SAS Institute, Cary, NC, USA), with macro period and treatments as the main effects, whereas buffaloes were considered as a random effect. The mathematical model was used for the analysis:


$${\text{Y}}_{ijklm}=\mu +{\text{Buff}}_{i}+{\text{Per}}_{j}+{\text{wk}}_{k}\;({\text{Per}}_{j})+{\text{fNDF}}_{l}+{\text{FT}}_{m}+{\text{fNDF}}_{l}\times {\text{wk}}_{k}({\text{Per}}_{j})+{\text{fNDF}}_{l}\times {\text{FT}}_{m}+{ɛ}_{ijklm}$$

where Y is the response variable (variable of interest), μ is the overall mean, Buff*_i_* represents the random effect of buffalo (*i* = 1 to 16), Per*_j_* represents the macro period of 6-wk (*j* = 1 to 2), wk*_k_* (Per*_j_*) represents the 3-wk subperiod (*k* = 1 to 2) within 6-wk macro periods, fNDF = fixed effect of fNDF (*l* = 1 to 2), FT*_m_* = fixed effect of fat (*m* = 1 to 2), fNDF*_l_*×FT*_m_* = interaction between PT and FT effects and ɛ*_ijklm_* = residual random error term. This model conferred considerable accuracy in the statistical test for the fat supply and limited the power of the fNDF effect because of the small number of degrees of freedom and the largest residual error used [16]. The values were reported as means with standard errors of the mean, and treatment differences were considered significant when p<0.05 and tendency was set as 0.05<p≤0.10.

## RESULTS

### Milk production, composition, body weight, body condition score, and rumen pH

An interaction trend was found between fNDF and dietary fat for milk fat content (p = 0.07) (Table 2). However, increasing the level of fNDF did not change the milk yield and components (p>0.10). Similarly, ECM, FCM, PCM yields, MkE, and MkN remained unaffected by the levels of fNDF supplied (p>0.10). There were no interactions (p>0.10) between fNDF and dietary fat levels for BW, body condition score (BCS), and rumen pH. Increasing the fNDF levels increased the BW, BCS, and rumen pH by 1.45%, 3.86%, and 1.78%, respectively (p≤0.02). Milk fat content was increased by 2.06% (p<0.01), and fat yield tended to increase by 2.58% (p = 0.06) with increasing dietary fat levels. The increasing levels of dietary fat supplies did not alter the contents and yields of lactose and protein (p>0.10). The FCM yield tended to increase by 2.05% (p = 0.10), whereas ECM, PCM, and milk yields remained unchanged by increasing the levels of dietary fat (p>0.10). Similarly, the levels of dietary fat did not affect the MkE and MkN (p>0.10). The BW was increased by 1.05% (p<0.01), whereas BCS and rumen pH remained unaffected with increasing dietary fat levels (p>0.10).

### Feed and production efficiencies

Increasing the levels of fNDF did not affect the feed efficiency (p>0.10) (Table 3). Similarly, MNE and MkN to milk energy ratio remained unaffected with increasing dietary fNDF levels (p>0.10). Gross and metabolic efficiencies of MP averaged 0.23 and 0.33, respectively in the dietary treatments. Milk nitrogen efficiency was increased by 3.43% (p<0.01), whereas the milk nitrogen (MkN) to milk energy ratio decreased by 1.34% by enhancing the dietary fat levels (p = 0.02).

### Blood metabolites

The glucose, cholesterol, and triglycerides concentration did not change (p>0.10) by fNDF levels (Table 4). The increasing levels of dietary fat increased the cholesterol concentration by 19.84% (p<0.01), whereas, the glucose and triglyceride remained unaffected (p>0.10).

### Predicted methane production

The increasing levels of dietary fat reduced the predicted methane production (CH_4_)_,_ whereas it increased (p<0.01) with increasing fNDF (Table 5). However, no interaction (p>0.10) was found between fNDF and fat for CH_4_. The high fNDF diet increased (p<0.01) the CH_4_ production (MJ) by 1.05%, (MCal) by 1.17%, (g/d) by 1.21%, CH_4_ yield (g/kg of DMI) by 1.03%, and CH_4_ intensity (gram/kg of MY) by 2.82% compared to low fNDF diet. Increasing the dietary fat levels reduced (p<0.01) the CH_4_ production (MJ) by 0.35%, (MCal) by 0.29%, (g/d) by 3.52%, CH_4_ yield (g/kg of DMI) by 0.55% and not affected the CH_4_ intensity (gram/kg of MY) (p = 0.32).

## DISCUSSION

### Milk yield was not affected by increasing forage-neutral detergent fiber and fat

Increasing the dietary fNDF levels from 23% to 33% did not increase the milk yield, as observed in this study and consistent with the findings of Alzahal et al [8] because similar DMI resulted in similar net energy for lactation intake, which directly influences milk yield [5]. Similarly, Piantoni et al [11] reported similar milk and FCM yields even with a difference of 2 kg DMI/d with low vs. high fiber diets (23.9 vs 21.9 kg/d), probably, because of a more energy partitioning towards milk fat content. In contrast to our findings, various studies documented reduced milk yield with increasing NDF supplies in cows [17,18].

In the current study, milk yield did not increase by dietary fat supplies and similar findings have been reported by previous studies [11,19]. Contrary to our findings, Hifzulrahman et al [2] and Anwar et al [20] reported increased milk yields with increasing fat levels in lactating buffaloes. This discrepancy may be explained by the use of early lactation buffaloes in their studies [2,20] where nutrient demand was a limiting factor for achieving higher milk production and thus the supplementation of energy in the form of bypass fat had a positive effect on milk production. In contrast, the buffaloes in our study were in mid-lactation, a stage where both production levels and nutrient demands were lower. It might be the reason for not getting any beneficial effect on milk production. Similar findings were observed in the study by Mudgal et al [21], which supports this explanation. The FCM yield tended to increase with dietary fat supplies in the present study due to high fat content which is similar to the findings of [2,20] but contradicted the findings of Piantoni et al [11].

### Milk fat content and yield were increased by increasing saturated fat

Increasing the dietary fNDF levels did not alter the milk fat content or yield in the current study, which is in contradiction with the findings of Piantoni et al [11] who found increased milk fat content on higher fNDF diets. Milk fat yield and content increased by dietary fat levels are in line with the previous studies of Hifzulrahman et al [2] and Anwar et al [20] in buffaloes. The direct association between dietary, plasma, and milk FA led to an enhanced milk fat in the fat added group. An increase in milk fat yield and content in the present study is likely due to the dietary incorporation of C16:0 into the milk fat, as the udder prefers to incorporate dietary C16:0 into milk fat than other fatty acids [22]. Contrary to our findings, a previous study by Ranjan et al [19] reported no response on milk fat content due to dietary fat. The possible reason was due to differences in treatment duration, feed type, and animal lactation stage.

### An interaction trend was observed between forage-neutral detergent fiber and fat for milk fat content

In the present study, an interaction trend was reported between fNDF and fat for milk fat content and these results are in line with the study of Benchaar et al [10] reported in dairy cattle. In the current study, the highest milk fat content was found in the high forage high fat treatment as the buffering capacity of the rumen was more due to high chewing activity owing to the more effective fiber in the high fNDF diet. This buffering capacity provided optimum pH for the FA to incorporate into milk fat even at increased levels of acetate production. However, Piantoni et al [11], Sterk et al [23], and Anwar et al [20] documented no interaction between fat and fNDF for milk fat content in lactating cattle or buffaloes, respectively.

### Body weight, body condition score, and rumen pH were increased by increasing forage-neutral detergent fiber

BW and BCS increased by fNDF, possibly due to high fiber degradation and increased acetate production in the rumen. The energy derived from acetate was likely directed towards body reserves, resulting in increased BW and BCS rather than increasing milk yield or milk fat in the current study. However, previous studies reported [13,18] no effect of increasing NDF on BW in cows. Furthermore, Piantoni et al [11] reported greater BW and BCS losses on higher fNDF diets in early post-partum lactating cows at ad-libitum feed intakes. The inconsistent effects of increased fNDF in the diets may be attributed to the energy status, which is controlled by the stage of lactation and the feed intake level in dairy animals. The energy partitioning for various metabolic functions such as milk or body gain is mainly controlled by the energy status of the animal [24].

In early to peak lactation, the lactating animals prioritize nutrient allocation towards milk and fat content production, as the transfer efficiency of plasma fatty acids to mammary tissue is greater than to the adipose tissue. This phenomenon was supported by the findings of Piantoni et al [11], where high fNDF diets increased milk fat contents and caused more body mobilization in the form of more BW and BCS losses. As lactation progresses, the transfer efficiency of plasma fatty acids to mammary tissue decreases, reflecting a shift in nutrient partitioning toward adipose tissue as the animal attains a positive energy balance. The buffaloes in the current study were in mid-lactation, indicating a more positive energy balance that favored nutrient allocation to adipose tissue formation, and increasing body reserves. BW was also increased by fat levels in this study, and these findings are in agreement with the study of Singh and Singh [25] in buffalo because additional energy provided through fat feeding was used for body fat deposition.

Ruminal pH indicates the balance between acid production and acid removal through neutralization and absorption in the rumen. Feeding a high F:C diet lowered acid production due to less starch fermentation in the rumen compared with a low F:C diet. High fNDF level resulted in higher ruminal pH in the present study and these findings are in line with the literature [13,17]. The addition of wheat straw into the diets possibly resulted in increased physically effective NDF, contributing to the stabilization of the rumen environment [17].

### Methane production decreased by increasing saturated fat

Methane production increased by increasing the dietary fNDF in the current study, and these findings are in agreement with Hassanat et al [26]. The ruminal methane production is directly linked with H_2_ formation [27] which is produced mostly by the fermentation of cell wall carbohydrates to butyrate and acetate [28]. Therefore, methane production can be decreased by depriving methanogens of H_2_. Higher fiber increases the production of acetate (an H_2_-liberating reaction). In contrast, Livingstone et al [29] reported no effect of forage type on the production of methane. Interestingly, methane production increased with high fNDF, as high fiber favored acetate production, which increased the H_2_ that was captured by the methanogens to produce methane. In the present study, increasing fat levels reduced methane production is in line with the results of Beauchemin et al [30].

### Cholesterol levels increased by feeding saturated fat

In the current study, the glucose, cholesterol, and triglyceride concentration did not alter by fNDF supplies. The levels of glucose and triglyceride did not change with dietary saturated fat supplies. These results are consistent with the study of fat feeding in buffaloes [19]. The glucose level did not change likely due to the body’s homeostatic mechanism, which prevents significant variation in glucose levels [31]. Cholesterol was increased by fat feeding in the present study similar to the findings of Ranjan et al [19] likely due to more uptake of fatty acid required for transporting the dietary fat. The other possible reason was likely due to the incorporation of long-chain fatty acids [31].

## CONCLUSION

The interaction between fNDF and dietary saturated fat supplies positively influences the milk fat content under restricted feeding conditions in mid-lactation buffaloes. The results indicate that feeding greater fNDF levels enhances BW and body condition score. The fat supplementation 2%/kg feed enhances milk fat and reduces methane production. Further studies are required to explore the interaction effects of fNDF and dietary saturated fat supplies on production responses in early lactating buffaloes.

## Figures and Tables

**Table 1:** The ingredient and chemical composition of diets

Ingredient (% of DM, unless noted)	Dietary treatments^1)^			

	23% fNDF		33% fNDF	
	
	0% Fat	2% Fat	0% Fat	2% Fat
Wheat straw	29.7	29.6	42.9	42.1
Corn grain	18.3	17.8	24.8	24.3
Molasses	1.84	1.79	2.57	2.52
Wheat bran	34.7	33.7	12.5	12.2
Canola meal	11.5	11.2	12.1	11.9
Soybean meal	1.92	1.86	3.05	2.99
Mineral mixture^2)^	1.02	0.99	0.83	0.81
Dicalcium phosphate	0.39	0.38	0.32	0.31
Salt (NaCl)	0.39	0.38	0.32	0.31
Urea (246%)	0.23	0.23	0.57	0.56
Saturated fat	-	2.11	-	2.07
Nutrient composition (% of DM)				
DM	90.2	90.4	90.7	90.9
Forage	29.7	29.6	42.9	42.1
CP	11.5	11.2	11.5	11.3
Ash	7.32	7.50	6.46	6.65
NDF	43.8	43.2	45.4	44.5
ADF	23.0	22.7	26.7	26.2
NFC^3)^	35.9	34.9	35.4	34.7
EE	3.43	5.44	2.99	4.98
Predicted nutritive value				
Forage NDF (%)	23.4	23.4	33.9	33.2
RUP (% CP)	31.0	31.1	30.5	30.6
RDP (% CP)	69.0	68.9	69.5	69.4
MP (g/kg)	91.0	88.7	86.1	84.3
ME (Mcal/kg)	2.35	2.50	2.20	2.35
NEL (Mcal/kg)	1.51	1.61	1.41	1.51
Starch (% of DM)	25.4	24.6	25.3	24.7
Sugar (% of DM)	5.39	5.24	5.14	5.03
RDP: RUP	2.23	2.19	2.28	2.27

1) Dietary treatments consisted of 4 diets with 2 levels of forage NDF (23.4% and 33.5% fNDF) and 2 levels of saturated fat (0% and 2% fat) on a DM basis in the diets. Saturated fat 300 g was added to high fat treatment whereas, low fat had no additional fat.

2) Mineral mixture was comprised of 0.7% DCP, 0.23% salt, 0.05% MgSO_4_, 0.007% FeSO_4_, 0.005% ZnSO_4_, 0.005 %MnSO_4_, 0.0013 %CuSO_4_, 0.001% CoCl, 0.005% KI.

3) NFC = 100–(CP+NDF+ash+EE).

fNDF, forage-neutral detergent fiber; NFC, non-fiber carbohydrates; DM, dry matter; CP, crude protein; NDF, neutral-detergent fiber; ADF, acid detergent fiber; NFC, non-fiber carbohydrate; EE, ether extract; RUP, rumen undegradable protein; RDP, rumen degradable protein; MP, metabolizable protein; ME, metabolizable energy; NEL, net energy for lactation.

**Table 2:** Milk production and composition of buffaloes fed with different fNDF and fat levels

Item	Dietary treatments^1)^				SEM	p-value^2)^		

	23%fNDF		33%fNDF					
		
	0% Fat	2% Fat	0% Fat	2% Fat		fNDF	Fat	fNDF×Fat
Milk (kg/d)	7.81	7.84	7.76	7.81	0.356	0.59	0.53	0.91
Yield (g/d)								
Fat	547	555	532	553	16.6	0.26	0.06	0.38
Protein	277	279	277	278	13.3	0.84	0.65	0.70
Lactose	375	375	373	374	17.9	0.62	0.87	0.99
Milk composition (%)								
Fat	7.10	7.15	6.96	7.20	0.167	0.40	<0.01	0.07
Protein	3.54	3.56	3.57	3.55	0.022	0.30	0.92	0.12
Lactose	4.79	4.77	4.79	4.78	0.031	0.88	0.28	0.95
ECM (kg/d)	11.8	11.9	11.6	11.8	0.42	0.36	0.13	0.57
4% FCM (kg/d)	11.3	11.5	11.1	11.4	0.38	0.31	0.10	0.47
3.4% PCM (kg/d)	6.64	6.67	6.61	6.70	0.629	1.00	0.66	0.81
MkE (Mcal/d)	8.12	8.21	7.98	8.17	0.288	0.36	0.14	0.58
MkN^3)^ (g/d)	43.3	43.7	43.4	43.5	2.08	0.84	0.65	0.70
BW (kg)	523	527	529	536	11.6	<0.01	<0.01	0.40
BCS	2.94	3.02	3.11	3.08	0.079	0.02	0.60	0.22
pH	6.77	6.73	6.87	6.87	0.016	<0.01	0.26	0.17

1) Dietary treatments consisted of 4 diets with 2 levels of forage NDF (23.4% and 33.5% fNDF) and 2 levels of saturated fat (0% and 2% fat) on a DM basis in the diets.

2) fNDF×Fat = interaction between fNDF and fat.

3) Milk N = milk true protein/6.38.

fNDF, forage-neutral detergent fiber; SEM, standard error of the mean; ECM, energy-corrected milk; FCM, fat-corrected milk; PCM, protein-corrected milk; MkE, milk energy; MkN, milk nitrogen; BW, body weight; BCS, body condition score.

**Table 3:** Production efficiencies of lactating buffaloes fed with different fNDF and fat levels

Item	Dietary treatments^1)^				SEM	p-value^2)^		

	23%fNDF		33%fNDF					
		
	0% Fat	2% Fat	0% Fat	2% Fat		fNDF	Fat	fNDF×Fat
Feed efficiency^3)^	0.56	0.56	0.55	0.56	0.025	0.56	0.64	0.97
ECM: DMI	0.84	0.85	0.82	0.84	0.030	0.42	0.12	0.77
4% FCM: DMI	0.81	0.82	0.79	0.81	0.027	0.28	0.13	0.54
3.4% PCM: DMI	0.47	0.48	0.47	0.48	0.045	0.95	0.69	0.88
MNE^4)^	16.7	17.3	16.7	17.2	0.82	0.63	<0.01	0.76
MkN: MkE^5)^ (g/Mcal)	5.32	5.31	5.42	5.29	0.093	0.15	0.02	0.06

1) Dietary treatments consisted of 4 diets with 2 levels of forage NDF (23.4% and 33.5% fNDF) and 2 levels of saturated fat (0% and 2% fat) on a DM basis in the diets.

2) fNDF×Fat = interaction between fNDF and fat.

3) Feed efficiency = milk yield/DMI.

4) MNE = (N in milk/N intake)×100.

5) MkE = milk nitrogen (g/d)/milk energy (Mcal/d).

fNDF, forage-neutral detergent fiber; SEM, standard error of the mean; ECM, energy-corrected milk; DMI, dry matter intake; FCM, fat-corrected milk; MNE, milk nitrogen efficiency; MkN, milk nitrogen; MkE, milk energy.

**Table 4:** Plasma metabolites of lactating buffaloes fed with different fNDF and fat levels

Item	Dietary treatments^1)^				SEM	p-value^2)^		

	23% fNDF		33% fNDF					
		
	0% Fat	2% Fat	0% Fat	2% Fat		fNDF	Fat	fNDF×Fat
Glucose (mg/dL)	83.5	81.0	88.7	84.6	2.70	0.11	0.23	0.77
Triglyceride (mg/dL)	123	128	127	125	2.1	0.77	0.45	0.13
Cholesterol (mg/dL)	109	129	104	126	3.5	0.30	<0.01	0.78

1) Dietary treatments consisted of 4 diets with 2 levels of forage NDF (23.4% and 33.5% fNDF) and 2 levels of saturated fat (0% and 2% fat) on a DM basis in the diets.

2) fNDF×Fat = interaction between fNDF and fat.

fNDF, forage-neutral detergent fiber; SEM, standard error of the mean.

**Table 5:** Predicted enteric methane production (CH_4_) of buffaloes fed with different fNDF and fat levels

Item	Dietary treatments^1)^				SEM	p-value^2)^		

	23% fNDF		33% fNDF					
		
	0% Fat	2% Fat	0% Fat	2% Fat		fNDF	Fat	fNDF×Fat
CH_4_^3)^ (MJ)	14.2	14.2	14.4	14.3	0.01	<0.01	<0.01	0.76
CH_4_^4)^ (Mcal)	3.40	3.39	3.44	3.43	0.003	<0.01	<0.01	0.76
CH_4_^5)^ (g/d)	238	238	241	240	0.76	<0.01	<0.01	0.76
CH_4_^6)^ (g/kg) DMI	17.0	16.9	17.2	17.1	0.01	<0.01	<0.01	0.67
CH_4_^7)^ (g/kg) Milk	31.5	31.1	32.3	32.1	1.38	<0.01	0.32	0.60

1) Dietary treatments consisted of 4 diets with 2 levels of forage NDF (23.4% and 33.5% fNDF) and 2 levels of saturated fat (0% and 2% fat) on a DM basis in the diets.

2) fNDF×Fat = interaction between fNDF and fat.

3) CH_4_ (MJ) = methane production in mega joule = (0.436+0.678×DMI+0.697×NDF intake).

4) CH_4_ (Mcal) = methane production in mega calorie = CH_4_, MJ/4.18.

5) CH_4_ (g/d) = methane production in gram per day = (0.671/40)×methane production in mega joule×1,000.

6) CH_4_ (g/kg DMI) = methane yield in gram per kg of DMI = CH_4_, g/d/ DMI.

7) CH_4_ (g/kg Milk) = methane intensity in gram per kg of milk.

fNDF, forage-neutral detergent fiber; NDF, neutral-detergent fiber; DMI, dry matter intake.
